# High return to sports and return to work rates after anatomic lateral ankle ligament reconstruction with tendon autograft for isolated chronic lateral ankle instability

**DOI:** 10.1007/s00167-022-06937-0

**Published:** 2022-03-31

**Authors:** Marco-Christopher Rupp, Hannes Degenhardt, Philipp W. Winkler, Maximilian Hinz, Yannick J. Ehmann, Andreas B. Imhoff, Jonas Pogorzelski, Alexander Themessl

**Affiliations:** 1 Department of Orthopaedic Sports Medicine, Technical University of Munich, Ismaninger Str. 22, 81675 Munich, Germany; 2 Alps Surgery Institute, Clinique Générale Annecy, 4 Chem. de la Tour la Reine, 74000 Annecy, France

**Keywords:** Lateral ankle instability, Ankle ligament reconstruction, Tendon autograft, Return to sports, Return to work

## Abstract

**Purpose:**

To evaluate clinical outcomes as well as return to sports (RTS) and return to work (RTW) rates following anatomic lateral ankle ligament reconstruction with a tendon autograft for chronic lateral ankle instability (CLAI) in a high-risk population, and to compare these outcome parameters between patients having received a gracilis tendon autograft (GT) and free split peroneus brevis tendon (PBT) autograft.

**Methods:**

Twenty-eight consecutive patients, who were diagnosed with CLAI, presenting with ≥ 1 risk factor (ligamentous hyperlaxity, insufficient substance of native ligament and/or high-demand athlete), who underwent ankle ligament reconstruction with a tendon autograft between January 2011 and December 2018, were included in this retrospective study. At 63.7 ± 28.0 months (24–112), 23 patients with a mean age of 29.7 ± 10.9 years were available for follow-up. The Karlsson Score, the Foot and Ankle Outcome Score (FAOS), the Tegner Activity Scale and the visual analog scale (VAS) for pain were collected at a minimum follow-up of 24 months. RTS and RTW were evaluated by questionnaire. A subgroup analysis with regard to the graft used for ankle ligament reconstruction (GT versus PBT) was performed.

**Results:**

Patients reported a Karlsson score of 82.1 ± 17.5 (37–100), a FAOS score of 87.8 ± 8.4 (73–99), a median Tegner activity scale of 5.0 (IQR 4–6) and a VAS for pain of 0.5 ± 0.9 (0–4) at rest and of 2.0 ± 2.1 (0–7) during activities at final follow-up. Postoperatively, 96% of patients had returned to sports after 8.3 ± 6.2 months. All patients (100%) had returned to work at 3.5 ± 5.7 (0–24) months, with 87% reporting an equal or improved working ability compared to that preoperatively. Postoperatively, exercise hours per week were significantly reduced compared to preoperatively in patients with a split PBT (*n* = 12; 13.0 ± 12.9 to 5.6 ± 6.4 h, *p* = 0.038) autograft as opposed to patients with a GT autograft (*n* = 11; 13.1 ± 8.7 to 12.4 ± 7.1 h, n.s.). No other group differences were observed.

**Conclusion:**

Good patient-reported outcomes as well as excellent RTS and RTW rates can be achieved in high-risk patients undergoing ankle ligament reconstruction with a tendon autograft for CLAI. These results may be helpful in preoperatively managing patients’ expectations regarding sports- and work-related outcomes and provide tangible data on the expectable time frame of the individual return to sports and work trajectory.

**Level of evidence:**

IV.

**Supplementary Information:**

The online version contains supplementary material available at 10.1007/s00167-022-06937-0.

## Introduction

Within sports-related injuries, supination ankle sprains with subsequent injury to the lateral ankle ligaments range among the most common musculoskeletal injuries, with a risk-profile dependent incidence of 2–58 per 1000 person years, accounting for 10–30% of all sports injuries [10, 45]. A growing body of evidence demonstrates that a surgical repair of the anterior talofibular ligament (ATFL) and the calcaneofibular ligament (CFL) via the Broström procedure [1] and its modifications [13, 24, 35] reliably produces favorable outcomes in cases of recurrent/chronic instability [18, 23, 31, 34, 47].

However, suboptimal outcomes have been reported in patient populations especially at risk due to high intensity athletic or occupational activities [11], generalized hyperlaxity [47], high-grade instability with insufficient substance of native ligament structures [2, 23], or in revision cases [19]. In these situations, surgical management with tendon graft-based anatomic lateral ankle ligament reconstruction has been advocated [37]. While there exists no surgical consensus to date concerning optimal fibular tunnel configuration and graft choice, both allograft- [5, 7, 8, 16, 21, 31, 34] as well as autograft-based techniques [3, 33] have demonstrated to reliably restore stability. With favorable clinical outcomes reported there remains a scarcity of evidence around sports and work-related outcomes [18]; an aspect paramount to individual postoperative satisfaction in young and physically active patients as well as for surgeons to better manage their patients’ expectations preoperatively.

Thus, the primary goal of this study was to evaluate clinical outcomes as well as return to sports (RTS) and return to work (RTW) rates following ankle ligament reconstruction with a tendon autograft for CLAI in a high-risk population. The secondary goal was to analyze the impact of graft choice used for ankle ligament reconstruction (gracilis tendon vs. split peroneus brevis tendon autograft) on these outcomes measures. It was hypothesized that RTS and RTW rates would be high, and that no significant difference in RTS, RTW, and clinical outcomes exists between procedures performed with GT and split PBT autograft at a minimum follow-up of 24 months.

## Materials and methods

**Fig. 1:**
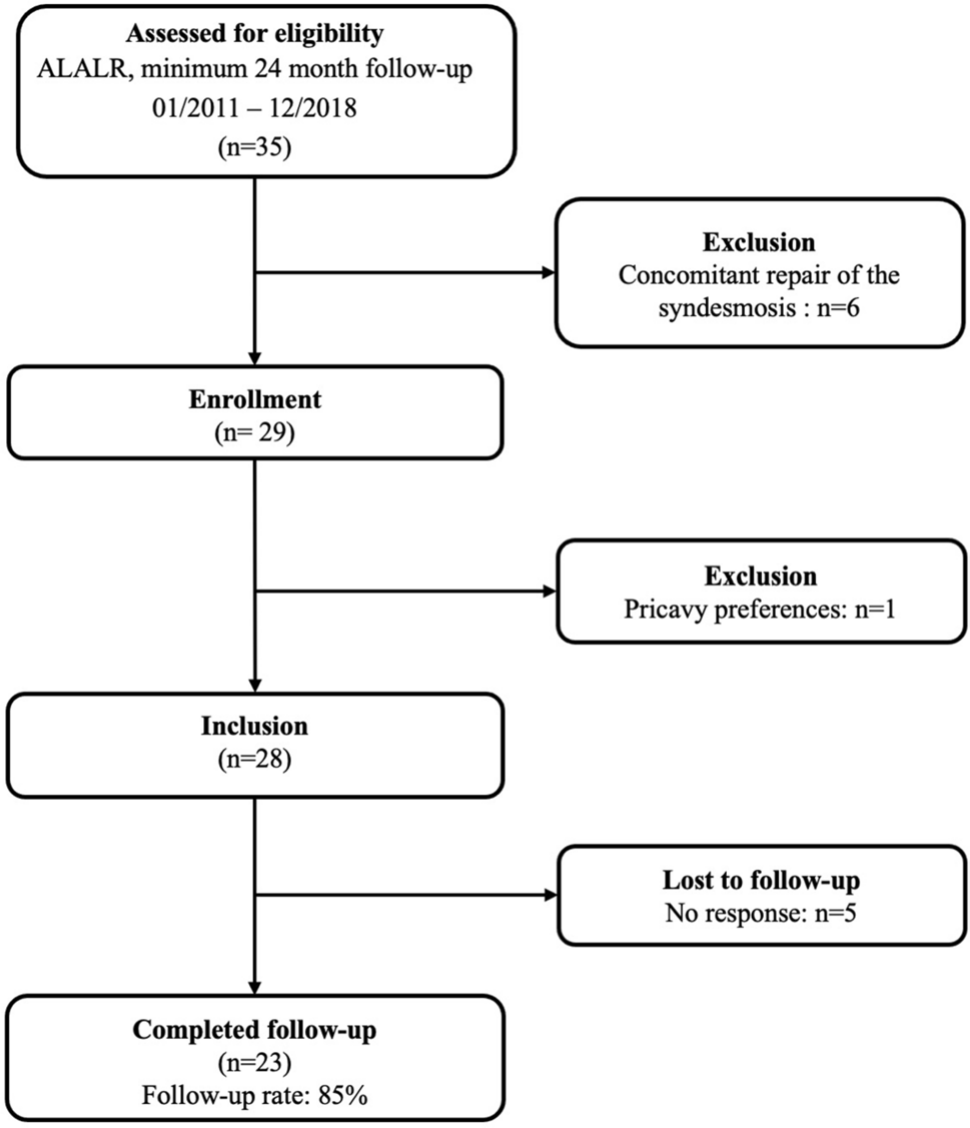
Flow chart visualizing the patient population for this study after accounting for inclusion criteria, exclusion criteria, and those lost to follow-up. *ALR* anatomic lateral ankle ligament reconstruction

This was a monocentric, Institutional-Review-Board (Technical University of Munich, ID number 262/20 S) approved retrospective case series. Review of the institutional data bank of the Technical University of Munich was performed to identify patients meeting the following inclusion criteria: patients who underwent ankle ligament reconstruction for the treatment of CLAI between January 2011 and December 2018 with a minimum follow-up of 24 months. Informed consent was obtained by each patient. Patients were excluded, if they underwent concomitant reconstructive surgery such as syndesmosis repair or scaffold-based cartilage regenerative surgery of the ipsilateral ankle at index surgery or reconstructive surgery at the ipsilateral lower extremity unrelated to the index surgery during the follow-up period or if they refused study participation for privacy preferences a priori. 35 patients who underwent ankle ligament reconstruction were identified. After applying inclusion and exclusion criteria as well as accounting for the patients lost to follow-up, twenty-three patients (10 men, 13 women; 82% follow-up) were included in the final data analysis, as detailed in Fig. 1. Comprehensive information on the patients demographic and surgical data is demonstrated in Table 1.

**Table 1:** Demographic and Surgical Data of the Study Population

Variable	Data
Sex	
Female, *n* (%)	13 (56.5)
Male, *n* (%)	10 (43.5)
Age^a^ (years)	29.7 ± 10.9 (17–51)
BMI (kg/m^2^)	26.1 ± 5.2 (18–36)
Smoker	
Yes, *n* (%)	1 (4.3)
No, *n* (%)	20 (87.0)
n.a., *n* (%)	2 (8.7)
Beighton Score	4.0 ± 2.4 (0–8)
Sports-related index trauma^b^	
Yes, *n* (%)	18 (78.3)
No, *n* (%)	5 (21.7)
Time between injury and diagnosis (months)	54.0 ± 80.1 (6–360)
Time between diagnosis and surgery (months)^c^	3.4 ± 3.2 (0.3–12)
Autograft	
Gracilis tendon, *n* (%)	11 (47.8)
Peroneus brevis tendon, *n* (%)	12 (52.2)
Concomitant procedures	3 (13.0)
Ganglion resection, *n* (%)	1 (4.4)
Osteophyte resection, *n* (%)	2 (8.7)

Continuous variables are presented as mean ± standard deviation (range); Categorical variables are presented as count and percentage.

*N.a*. not available, *BMI* body mass index

^a^Age at surgery

^b^Index trauma, which led to chronic lateral ankle instability

^c^Timing of surgery depended on whether the patient had undergone adequate conservative treatment prior to first presentation at our institution

### Indication

For the purpose of this study, the following evidence-based criteria were required for the diagnosis of CLAI [14]: (1) A history of the previously injured ankle joint “giving way”, and/or recurrent sprain and/or subjective “feelings of instability”, (2) objective insufficiency of ATFL and CFL in the clinical assessment via the anterior drawer test and the talar tilt test, and (3) symptoms for a time period greater than 6 months. In clinical suspicion of concomitant injuries and to assess ligament structure, magnetic resonance imaging was performed preoperatively. ankle ligament reconstruction was indicated in patients with CLAI who were refractory to non-operative treatment consisting of targeted physiotherapy focused on proprioception and peroneal strengthening for a minimum of 3 months [2] or in revision cases after failed direct ligament repair. Furthermore, for the classification as a “high-risk” case, the presence of one of the following risk factors was required: generalized ligamentous hyperlaxity, insufficient soft tissue and/or high-demand athletes (participation in pivoting sports and/or athletic activity of more than 60 min, at least 3 week) [37]. For assessment of general laxity, the Beighton score was collected, with values ≥ 4 indicating generalized hyperlaxity [42]. Contraindications for anatomic lateral ankle ligament reconstruction were severe obesity (> 40 kg/m^2^), diabetic foot syndrome or vascular disease.

### Surgical technique

**Fig. 2:**
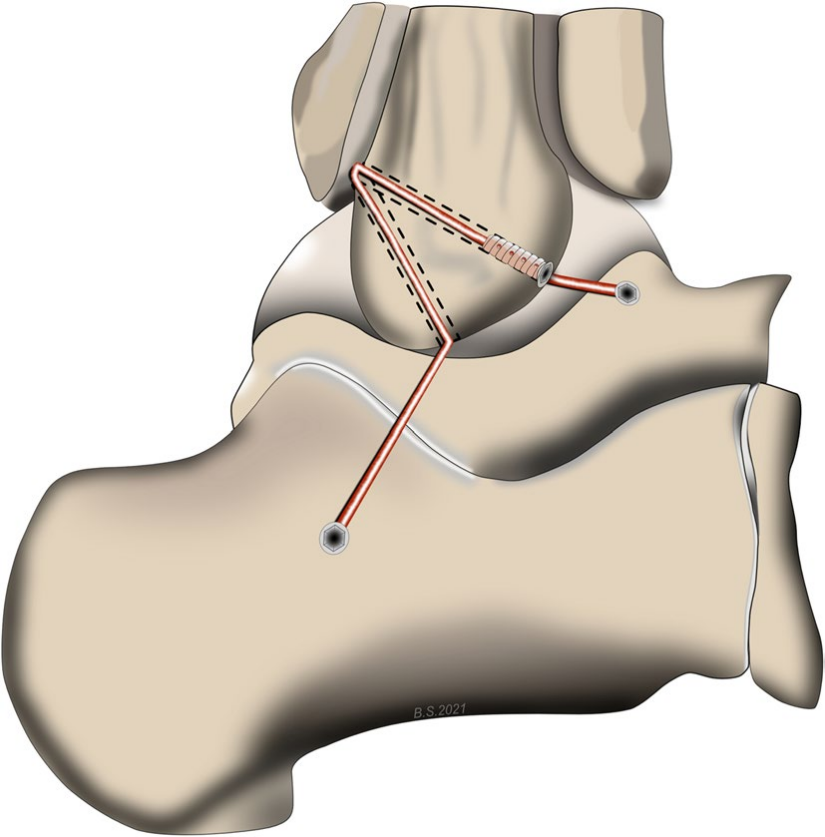
Anatomic lateral ankle ligament reconstruction technique. (1) The autograft, fixated in a drill hole at the talar insertion site of the ATFL with an absorbable anchor, is shuttled anterior-posteriorly through a fibular tunnel at the fibular insertion site of the ATFL and fixated at the anterior tunnel aperture with an absorbable anchor. (2) The graft is shuttled posteriorly-anteriorly through a second fibular tunnel at the origin of the CFL and fixated into a drill hole at the calcaneal insertion site of the CFL in 0° plantar flexion and 10° eversion with an absorbable anchor

Ankle ligament reconstruction was performed as previously described [6] and is illustrated in Fig. 2. After incision, the ligament quality (ATFL and CFL) was assessed to confirm the indication for ankle ligament reconstruction. Subsequently two 4 mm tunnels were convergently placed in V-shaped configuration into the fibula at the insertion areas of the ATFL and CFL and a shuttle suture (FiberLink, Arthrex Inc., Naples, Florida, USA) was introduced. Next, a 4.5 mm drill hole with a depth of 20 mm was placed at the talar insertion site of the ATFL, in which one end of the graft was fixated using a bioabsorbable anchor (4.75 mm PEEK SwiveLock^®^, Arthrex Inc., Naples, Florida, USA). After shuttling the graft through the fibular tunnels, the ATFL was reconstructed in 0° plantar flexion and 10° eversion, under tension of the graft by inserting an absorbable anchor (4.75 mm PEEK SwiveLock, Arthrex Inc., Naples, Florida, USA). Next, the graft was shuttled below the peroneal tendons and introduced into at 5 mm tunnel at the anatomic insertion site of the CFL at the calcaneus. CFL tenodesis was performed by fixating the graft in 0° plantar flexion and 10° eversion using an absorbable anchor (5.5 mm PEEK SwiveLock^®^, Arthrex Inc., Naples, Florida, USA) or 6 mm interference screw (e.g. 6 mm FastThread™-BioComposite Screw, Arthrex Inc., Naples, Florida, USA) depending on bone quality.

The graft choice (GT or PBT) was randomized to the individual preference of the surgeon. Harvest and preparation of the PBT graft was performed as previously described [6]. The gracilis tendon was harvested at the pes anserinus in a standard technique and prepared as described for the PBT graft.

### Postoperative rehabilitation

Patients were immobilized in a walker (protect.CAT Walker, medi Gmbh, Bayreuth, Germany) for 6 weeks. No weight-bearing was allowed for the first 2 weeks, and then gradually increased until full weight-bearing was permitted after 6 weeks. While flexion and extension were permitted during physiotherapy, pro- and supination was prohibited for 6 weeks postoperatively. Free active range of motion was permitted thereafter, with return to low-impact sports being allowed 3 months postoperatively and return to pivoting sports being allowed 6 months postoperatively.

### Clinical evaluation

Clinical outcome measures included the visual analog scale for pain (VAS) at rest and during activities, the Karlsson Score [26], the Foot and Ankle Outcome Score (FAOS) [40] and the Tegner Activity Scale [43], which were collected at a minimum follow-up of 24 months postoperatively.

### Return to sports

In the absence of validated return to sports questionnaires after lateral ligament reconstruction, patients were asked to complete a comprehensive RTS survey, which was developed based on previously published investigations evaluating RTS after lower extremity surgery [15, 38]. Patients were asked to detail their specific preinjury (prior to the injury warranting surgery) and postoperative (final follow-up) participation in sporting activities. Participants were asked to specify the frequency of participation (times per week), the level of sports (recreational, competitive, or professional) and the duration of each session (in hours).

On the basis of previously proposed categorizations [17], the respective sports disciplines were classified as “pivot” and “non-pivot” sports according to the frequency of cutting or pivoting moves of the ankle required, by two orthopedic sports medicine physicians in training (M.C.R. and A.T.) and in case of disagreement, consensus was achieved by consulting a third rater (H.D.) Furthermore, timing of partial RTS (RTS to any level of sports postoperatively), the timing of return to the current level of sports and a qualitative change of sporting ability (ordinal scale consisting of “improved”, “equal to preinjury level”, or “deteriorated”) were questioned. Next, patients were asked to rate their current sports-related function of the ankle (excellent, good, satisfactory, bad) and indicate, if they had received further surgery during follow-up. Finally, the patients were asked to specify potential donor site specific deficits (weakness of the knee, instability of the knee, problems with flexion or pronation of the foot, loss of sensitivity and options to self-indicate).

### Return to work

Participants were asked to provide details on their occupation (employment, self-employed, housework, retired, unemployed) and working hours per week (0, 0–10, 10–20, 20–30, 30–40, > 40), both prior to injury leading to surgery and at final follow-up. Subsequently, patients were questioned to classify the physical strain of their occupation according to the classification of the REFA association (classification by defined criteria into: occupation without specific physical strain or with either small, moderate, hard or most heavy physical strain) prior to injury leading to surgery and at final follow-up, as previously described in the setting of lower extremity surgery [41]. The time to RTW and recovery to full current physical working ability as well as time of postoperative sick leave were quantified. Finally, patients were asked to qualitatively assess the change of working ability (ordinal scale consisting of “improved”, “equal to preoperative state”, or “deteriorated”). To minimize the risk of recall bias, medical records were independently cross-referenced for RTW and RTS information to ensure adequacy of reporting and patients were contacted for clarification in case of discrepancies.

### Subgroup analysis

While the limited sample size limits the ability to draw strong conclusions, a subgroup analysis of two groups (patients undergoing ankle ligament reconstruction with GT vs. split PBT autograft) was performed to determine an influence of the tendon autograft choice on the postoperative outcome. Constitutional factors at baseline (BMI, age, sex, Beighton score) and follow-up intervals were compared between the two groups to exclude potential significant non-equivalence of the groups. The size of the study population statistically limited the number of outcome parameters to be evaluated, since repeatedly testing an excessive number of factors on a single dataset predisposes for the occurrence of Type 1 errors. Thus, the following variables were selected a priori, representing a selection of clinically meaningful primary outcome measures: Operative time, patient-reported outcomes scores as well as timing of RTS, number of sports and exercise hours per week, including potential pre- to postoperative changes of these parameters.

### Statistical analysis

Statistical analysis was performed using SPSS software version 26.0 (IBM-SPSS, New York, USA). The Shapiro–Wilk-test was performed to assess the distribution of continuous variables. Continuous variables were reported as mean ± standard deviation (range) or median (range) depending on the distribution. For unrelated group comparisons, continuous variables were analyzed employing a parametric unpaired *t* test or the non-parametric Mann–Whitney *U* test, while group comparison of categorical variables was performed with the Chi-square test or the Fisher’s exact test according to their respective distribution. Categorical variables were compared performing the binary Fisher’s exact test or the Chi-square test, as statistically appropriate. The parametric paired *t* test or the non-parametric Wilcoxon test for two related samples was used to compare pre- and postoperative continuous parameters while the sign test was applied for pre-to postoperative comparisons of categorial parameters. The level of significance was set at *p* < 0.05. A total sample size of 20 subjects was determined in an a priori power analysis, performed with G*Power (Erdfelder, Faul, Buchner, Lang, HHU Düsseldorf, Düsseldorf, Germany) [9] to achieve a statistical power of 0.8, to detect the minimal clinically important difference of 3.5 of the primary outcome measure, the FAOS sports subscale [44] between the graft subgroups for a two-sided t test for independent samples using a significance level of 5%. A within-group standard deviation of 2.5 points was assumed, translating to an effect size of *d* = 1.4. The post-hoc power analysis between the graft subgroups–though a monotonic transformation of the p-value of the FAOS sports subscale–detected a power of 0.09.

## Results

### Clinical outcomes

The outcomes scores were collected at a final follow-up of 63.7 ± 28.0 months (24–112). At final follow-up, patients indicated a mean VAS of pain score of 0.5 ± 0.9 (0–4) at rest and 2.0 ± 2.1 (0–7) during activities. Patients reached a Karlsson score of 82.1 ± 17.5 (37–100) and an overall FAOS score of 87.8 ± 8.4 (73–99) as well as a median score of 5.0 (IQR 4–6) on the Tegner activity scale. Detailed information on the subcategories of the FAOS score is listed in Table 2.

**Table 2:** Results of FAOS Subscales

FAOS subscales	Score
Pain	90.7 ± 11.2 (61–100)
Symptoms	78.3 ± 18.2 (39–100)
ADL	96.2 ± 4.7 (84–100)
Sports	83.9 ± 17.9 (45–100)
QOL	70.2 ± 17.9 (25–100)

Follow-up scores of different FAOS subscales. Continuous variables are presented as mean ± standard deviation (range)

*FAOS* foot and ankle outcome score, *ADL *activities of daily life, *QOL* quality of life

### Return to sports

All patients available for analysis (*n* = 23, 100%) actively participated in sports prior to their index trauma leading to CLAI, with 21 (91.3%) participating in pivoting sports. At final follow-up, 22 patients (96%) had returned to sporting activities in general and 19 (82.6%) had returned to pivoting sports. The mean time to a partial RTS was 8.3 ± 6.2 (1–24) months, while the mean time for reaching the current level of sports was 11.1 ± 8.0 (3–36) months. With regard to postoperative RTS rates, there were no statistically significant differences compared to preoperatively in the total number of sporting activities (3.6 ± 2.2 vs. 3.8 ± 1.8; n.s.), the number of pivoting sports performed (2.0 ± 1.3 vs. 1.7 ± 1.1, n.s.), or in the number of hours of sports exercised per week (12.4 ± 11.1 vs. 8.7 ± 7.4; n.s.). No significant pre- to postoperative changes in the sports level were observed (n.s.).

Overall, 20 patients (89.6%) reported an “excellent” (*n* = 4; 17.4%) or “good” (*n* = 16; 69.6%) sport-related function of the affected ankle, three patients (10.4%) rated the function of their affected ankle to be “satisfying” (*n* = 2; 8.7%), while one patient (4.3%) rated the ankle function as “bad”.

### Return to work

Postoperatively, all patients in this case series returned to work (*n* = 23; 100%). Patients returned to work at a mean of 6.7 ± 8.1 weeks (0–36), while a return to pre-injury level at work was achieved at a mean of 3.5 ± 5.7 months (0–24). Twenty patients (87%) reported either an “improved” (*n* = 5; 22%) or “equal” professional performance (*n* = 15; 65%) compared to preoperatively, while three patients (13%) reported a “deteriorated” working ability following surgery. Of the patients who reported a “deteriorated” performance, one patient had to change occupation and one patient had to transfer to a less strenuous activity at his workplace. Of note, all four patients reporting “most heavy” strain at work preoperatively had to change to a medium (*n* = 2) or light (*n* = 2) physical load at final follow-up. Compared to preoperatively, the type of employment and the working hours remained at a comparable level (n.s.) (Table 3).

**Table 3:** Categorical variables are presented as count and percentage; analysis of the study population included in the return to work analysis (*n* = 23)

Variable	Preoperatively	Postoperatively	*P* value
Type of work			n.s
Employed, *n* (%)	13 (57%)	15 (65%)	
Self-employed, *n* (%)	3 (13%)	3 (13%)	
Household, *n* (%)	0 (0%)	0 (0%)	
Retired, *n* (%)	0 (0%)	0 (0%)	
Unemployed, *n* (%)	1 (4%)	0 (0%)	
Student, *n* (%)	6 (26%)	5 (22%)	
Physical load			n.s
No strain, *n* (%)	5 (22%)	5 (22%)	
Small strain, *n* (%)	10 (44%)	10 (44%)	
Moderate strain, *n* (%)	3 (13%)	4 (17%)	
Hard strain, *n* (%)	1 (4%)	4 (17%)	
Heavy strain, *n* (%)	4 (17%)	0 (0%)	
Working hours/week			n.s
0	1 (4%)	0 (0%)	
0–10	2 (9%)	2 (9%)	
10–20	2 (9%)	1 (4%)	
20–30	2 (9%)	3 (13%)	
30–40	6 (26%)	6 (26%)	
> 40	9 (39%)	11 (48%)	

### Subanalysis with regard to graft choice

Out of the 23 patients included, eleven patients (48%) underwent anatomic lateral ankle ligament reconstruction with a gracilis tendon graft, while a split peroneus brevis tendon graft was used in twelve patients (52%). Baseline parameters including age, sex, BMI, Beighton score, and operative time were evenly distributed between both groups (n.s., respectively). Patient reported outcome scores including the VAS, Karlsson score, Tegner activity scale, and FAOS did not differ significantly between patients with GT autograft and patients with PBT autograft (supplementary table 1).

With regard to RTS parameters neither the time to RTS nor the number of sports performed postoperatively differed between groups (n.s., respectively). The exercise hours per week dedicated to sports significantly decreased in patients with PBT autografts (13.0 ± 12.9 to 5.6 ± 6.4 h, *p* = 0.038), while its did not reach statistical significance in patients with GT autografts (13.1 ± 8.7 to 12.4 ± 7.1 h, n.s.).

However, these results have to be interpreted with caution, as post-hoc power analysis revealed the sample size may be underpowered for this subgroup analysis (1-ß error probability = 0.09).

## Complications

Three patients of the GT group (27%) and two of the PBT group (17%) reported loss of sensibility in the scar region. None of the patients with GT autografts reported the sensation of instability or weakness of the knee with regard to the donor site of the tendon graft. By the time of final follow-up at a mean of 63.7 ± 28.0 months (24–112), one patient (4%) had undergone surgery of the ipsilateral foot, due to a calcaneal heel spur.

## Discussion

The most important finding of this study was that high-risk patients suffering from CLAI undergoing ankle ligament reconstruction with a tendon autograft demonstrated good clinical outcomes and high RTS and RTW rates. The findings of this study may be of clinical relevance, as tendon autograft-based ankle ligament reconstruction could be an effective surgical option in returning high-risk patients to sports and work at a low complication and revision rate at mid-term follow-up. These results may be helpful in preoperatively managing patients’ expectations regarding sports- and work-related outcomes.

Regarding the external validity of the findings, the demographic characteristics of the study population are comparable to previously described high-risk populations undergoing ankle ligament reconstruction with tendon grafts, with a Beighton score of 4.0 ± 2.4, participation in 3.8 ± 1.8 sport disciplines for 8.7 ± 7.4 h per week and 78.3% of the patients indicating the index trauma to be sports-related [7, 8, 22].

Regarding the clinical efficacy of ankle ligament reconstruction with a tendon autograft, the results of this study underscore the positive effect of the procedure, with 89.6% of the patients indicating good or excellent ankle function in the absence of systemic complications. The patient-reported outcome measures fall within range of previous studies following comparable graft-based stabilization surgery in high-risk patient populations, reporting Karlsson scores of 82.3–90.3 [7, 8, 22, 28] and FAOS total scores of 79.4–89.0 points, FAOS symptoms scores of 76.9–80.4 points, FAOS ADL scores of 91.9–93.4 points, FAOS sports scores of 75.0 points, FAOS QoL scores of 68.1 points [7, 8, 22, 28]. In patient populations not qualifying as high-risk, postoperative Karlsson scores as high as 95.0–95.3 points [3, 33]. with VAS ranging between 0.1 and 1.9 [3, 5, 22] and FAOS pain scores of 84.9–85.2 points [3, 33] have been reported following comparable autograft-based ankle ligament reconstruction.

The results of this study also have to be compared to outcomes following alternative techniques performing internal bracing by suture tape augmentation. While the patient populations and surgical indication in these studies slightly differ, the reported clinical outcome, with a total FAOS score ranging from 91.7 ± 7.7 to 93.7 ± 6.0 [30, 46], indicates that suture tape bracing may be a viable alternative.

Regarding the return to athletic activity, the findings of the present study fall within range of previous studies, reporting Tegner activity levels of 4–6 following ankle ligament reconstruction [5, 31, 34]. While evidence in the literature is scarce [18], RTS rates reported following allograft-based ankle ligament reconstruction techniques vary between 64 and 97% [5, 8, 16], resulting in pooled RTS rates of 80% [32]. Regarding the timing of RTS postoperatively, Ibrahim et al. [20] reported a mean RTS at 6.8 months following ankle ligament reconstruction with a gracilis autograft, while Coughlin et al. [3] reported RTS at a time point of 6.5 months postoperatively following ankle ligament reconstruction with a GT allograft; comparable to the results observed in the present study. Similarly, previous study observed difficulties to return to pre-injury level of sports following ankle ligament reconstruction [3, 5]

While comparability is limited due to differences in prevalence of risk factors in the patient populations, results have to be benchmarked against the Broström repair technique and its modifications as the gold standard [2, 5, 12]. Heterogenous rates of RTS to previous level of sports rates ranging between 20 and 100% have been described following Broström repair, resulting in a pooled rate of 85% at a mean of 4.7 ± 1.5 [18]. Studies directly comparing activity level and function between Broström repair and graft-based reconstruction techniques in a monocentric study design did not find significant differences [31, 34]. When comparing the results of arthroscopically-assisted internal bracing procedures, these report a heterogenous time frame of RTS ranging from 13.3 ± 5.2 weeks [29] to 170.7 ± 66.4 days depending on the postoperative rehabilitation [4].

With a scarcity around evidence regarding work-related outcomes in the contemporary, the results of this investigation seem to be comparable to 6.0–8.5 weeks reported after anatomic ligament repair without graft augmentation [25, 27].

The ability to derive strong clinical conclusions of the subgroup analysis comparing ankle ligament reconstruction performed with GT versus split PBT graft is limited by the sample size of this study. Yet, comparable to the results of our subgroup analysis, that did not show any significant differences in the patient-reported outcomes scores, Ramdass et al. [39] did not detect any differences in patient-reported outcomes in a comparison of 36 cases performed with a split PBT graft and 28 cases with ST allograft in a different surgical technique. However, the finding that undergoing ankle ligament reconstruction with a split PBT graft is associated with returning to significantly fewer exercise hours may be attributed to the weakening of the peroneus brevis in its biomechanically important role as an active stabilizer against supinatory forces [36]. While studies with higher statistical power are required to ultimately guide surgical decision making, the data may suggest performing ankle ligament reconstruction with a GT rather than PBT graft in high-risk patients.

In terms of practical clinical relevance, these results are helpful in preoperatively managing patients’ expectations concerning expectable clinical outcome and recovery, as tendon autograft-based ankle ligament reconstruction was observed to be an effective surgical option in returning to athletic and professional activity at a low complication and revision rate at mid-term follow-up and provides tangible data on the expectable time frame of the return to sports and work trajectory.

The findings of this investigation must be interpreted within the context of the study’s limitations. First, due to the lack of validated outcome measures for RTS and RTW in the setting of CLAI, questionnaires were designed similar to previous comprehensive investigations on work- and sports-related outcomes after lower extremity surgery [15, 38]. Second, with sports- and work-related patient-reported outcomes elected as primary endpoint, no comparison of pre- to postoperative patient-reported outcome scores was reported. Third, follow-up clinical examination and stress-radiographs to evaluate passive stability were not included, as this exceeded the scope of this study focused on subjective sports- and work-related patient-reported outcomes. Fourth, while comparable to previous outcome studies on ALankle ligament reconstruction techniques [5, 21], the sample size of this study was relatively small due to the strict inclusion criteria. Fifth, post-hoc power analysis revealed the the study cohort may be underpowered for a subgroup analysis with an 1-ß error probability of 0.09. Sixth, regarding the non-comparative aspect of treating patients in a single reference center for chronic ankle instability, the external validity of the results may be limited. Finally, as randomization of graft choice was performed according to the preference of the surgeon, a potential selection bias could not be excluded.

## Conclusion

Good patient-reported outcomes as well as excellent RTS and RTW rates can be achieved in high-risk patients undergoing ankle ligament reconstruction with a tendon autograft for CLAI. These results may be helpful in preoperatively managing patients’ expectations regarding sports- and work-related outcomes and provide tangible data on the expectable time frame of the individual return to sports and work trajectory.

## Supplementary Information

Below is the link to the electronic supplementary material.

Supplementary file1 (DOCX 16 KB)

## Abbreviations

ADL: Activities of daily life
ATFL: Anterior talofibular ligament
BMI: Body mass index
CFL: Calcaneofibular ligament
CLAI: Chronic lateral ankle instability
FAOS: Foot and ankle outcome score
GT: Gracilis tendon
N.a.: Information not available
n.s.: Not significant
PBT: Peroneus brevis tendon
QOL: Quality of life
RTS: Return to sport
RTW: Return to work
VAS: Visual analog scale
