# Supplementary material for: High return to sports and return to work rates after anatomic lateral ankle ligament reconstruction with tendon autograft for isolated chronic lateral ankle instability
Source: Knee Surg Sports Traumatol Arthrosc. 2022 Mar 31;30(11):3862–70. doi: 10.1007/s00167-022-06937-0 (PMC9568480; doi:10.1007/s00167-022-06937-0)
Supplement: Supplementary file 1 — Supplementary file1 (DOCX 16 KB) [file 167_2022_6937_MOESM1_ESM.docx]

| **Supplementary Table 1** |  | |  |
| --- | --- | --- | --- |
| **Variable** | **Graft** | | **P value** |
|  | GT (n=11) | PBT (n=12) |  |
| **Demographic data** | |  | 0.510 |
| Male *n (%)* | 4 (36%) | 6 (50 %) |  |
| Female *n (%)* | 7 (64%) | 6 (50 %) |  |
| ^a^Age (years) | 29.8 ± 10.7 | 29.6 ± 11.6 | 1.000 |
| BMI (kg/m^2^) | 26.3 ± 6.5 | 25.9 ± 4.1 | 0.854 |
| Beighton Score | 4.3 ± 2.4 | 3.6 ± 2.4 | 0.540 |
| **Surgical data** |  |  |  |
| Follow-up *(months)* | 57.6 ± 25.0 | 69.3 ± 30.5 | 0.325 |
| **Clinical outcomes** | |  |  |
| VAS | 2.4 ± 2.7 | 1.6 ± 1.5 | 0.662 |
| Karlsson Score | 76.6 ± 17.6 | 87.5 ± 16.3 | 0.092 |
| Tegner activity scale | 4.0 (3-10) | 5.0 (2-7) | 0.992 |
| FAOS total | 87.3±8.9 | 88.2±8.2 | 0.806 |
| **Return to Sports** | |  |  |
| Time to full RTS (months) | 10.1 ± 5.3 | 12.2 ± 10.3 | 1.000 |
| Sports practiced preoperatively, n (%)  Sports practiced postoperatively, n (%) | 3.6 ± 1.9  3.9 ± 1.7 | 3.5 ± 2.6  3.7 ± 1.9 | 0.685  0.814 |
| Activity preoperatively (hours/week)  Activity postoperatively (hours/week) | 13.1 ± 8.7  12.4 ± 7.1 | 13.0 ± 12.9  5.6 ± 6.4 | 0.975  0.084 |

***Supplementary data (table 1)****. Continuous variables are presented as mean ± standard deviation; Categorical variables are presented as count and percentage;*

*BMI, body mass index; VAS, visual analog scale; FAOS, Foot and Ankle Outcome Score; RTS, return to sports; RTW, return to work*

*^a^ Age at surgery*

*^*^ Statistically significant difference between groups (level of significance, p<0.05)*
